# Two-step regression analysis approach to assess burn wound severity using spatial frequency domain imaging

**DOI:** 10.1117/1.JBO.31.1.016005

**Published:** 2026-01-20

**Authors:** Thinh Phan, Christopher A. Campbell, Gordon T. Kennedy, Nicole Wakida, Nataliya Makeeva, Gabriela Tabone, Theresa L. Chin, Victor C. Joe, Bernard Choi, Anthony J. Durkin

**Affiliations:** a University of California – Irvine, Beckman Laser Institute and Medical Clinic, Irvine, California, United States; b University of California – Irvine, Department of Biomedical Engineering, Irvine, California, United States; c University of California – Irvine, Department of Surgery, Irvine, California, United States

**Keywords:** spatial frequency domain imaging, burn wound severity assessment, linear regression, logistic regression, collagen, picrosirius red, machine learning

## Abstract

**Significance:**

Prompt care is essential for burn wound recovery. Spatial frequency domain imaging (SFDI) has previously shown promise in predicting healing outcomes across burn severity grades. This study builds on that by demonstrating calibrated reflectance images ($R_{d}$) from SFDI can estimate thermally induced collagen denaturation depth (CDD), a histology-based metric of burn severity linked to healing outcomes. These findings may simplify future hardware design by clarifying contrast sources in SFDI.

**Aim:**

To develop predictive models for: 1) Day-1 postburn CDD using SFDI $R_{d}$ and 2) Day-28 healing outcomes using day-1 CDD.

**Approach:**

Using a previously reported graded-severity porcine burn model (n=4) with eight contact durations (5 to 40 s), we collected SFDI and color images on days 0, 1, 3, 7, 14, 21, and 28. Histological analysis using Picrosirius red staining and polarization microscopy was performed on days 1, 7, 14, 21, and 28 to assess CDD. Healing outcomes were clinically evaluated on day 28. For analysis, a two-step regression framework was applied:

Step 1: Multiple linear regression, where day-1 SFDI R_d_ is used to predict same-day CDD.

Step 2: Logistic regression, where day-1 CDD is used to predict day-28 healing outcome.

Together, these steps established a regression framework to predict day-1 CDD and day-28 healing outcomes using day-1 SFDI R_d_.

**Results:**

The linear model using $R_{d}$ across eight wavelengths (471-851 nm) and five spatial frequencies (0 to $0.2\;{\mathrm{mm}}^{-1}$) predicted CDD with a root mean square error of 105  μm and adjusted $R^{2}$ of 0.71. The logistic model predicted healing outcomes with an ROC-AUC of 0.88, supporting CDD as an early indicator for burn severity assessed by healing potential.

**Conclusions:**

This two-step framework enables early prediction (as early as day 1) of burn severity and healing using SFDI $R_{d}$.

## 1 Introduction

Thermal burns are dynamic wounds that can progress in terms of severity over the first 72 h.^1^^–^^4^ Timely triage and appropriate care during this period can help to optimize healing and minimize scarring, contractures, and infections. Burn wound severity assessment is clinically based on estimating the depth of tissue damage. Superficial-partial-thickness (SPT) burns, with damage contained within the upper half of the dermis, can heal with a satisfactory cosmetic outcome through daily topical wound care, which may include extended-wear dressing. Deep-partial-thickness (DPT) and full-thickness (FT) burns, with damage extending to most of or nearly the entire dermis (to the extent that the vascular supply is completely compromised), typically require operative excision and grafting.^5^^,^^6^ Experienced burn-oriented physicians can typically correctly identify and differentiate SPT and FT burns. However, distinguishing deeper SPT from DPT burns remains difficult even for experienced clinicians. Up to 40% of skilled burn surgeons struggle to differentiate SPT from DPT based on bedside assessment accurately.^7^^–^^9^ This impacts the trajectory of burn patient care, with implications for quality of healing outcomes, patient experience, and length of hospitalization.

Efforts to reduce subjectivity in burn severity assessment have focused on the development and evaluation of technologies that can measure compromised hemodynamics and physiological responses to the initial thermal insult. For example, laser doppler imaging (LDI), first used in this context in the early 1980s, has extensive literature support for assessing burn wounds by measuring perfusion and visualizing compromised superficial microvascular flow.^7^^,^^10^^–^^13^ However, LDI accuracy in burn severity assessment is not compelling within 24 to 48 h postinjury due to vasoconstriction related to thermal insult, as well as limited penetration depth. LDI also requires relatively long scan times, which introduces motion artifacts in burn patients who are typically prone to constant movements.^10^^–^^12^ In recent years, research efforts involving coupling machine learning (ML) algorithms with multispectral (MSI) and hyperspectral imaging (HSI) data to assess burn severity have gathered increasing interest.^14^^–^^18^ The spectral contrast observed in MSI and HSI relates to changes in tissue reflectance that correspond to functional changes, such as vessel coagulation leading to a reduction in metabolic activities, conversion of hemoglobin species, and edema. Although certain studies have demonstrated that scattering properties can be inferred from MSI and HSI data, these methodologies often depend on specific assumptions,^19^^–^^21^ given that unstructured (i.e., planar) reflectance measurements exhibit limited sensitivity to variations in scattering, particularly in scenarios such as collagen denaturation induced by thermal injury.^22^^–^^24^

Spatial frequency domain imaging (SFDI) is a noncontact, wide-field imaging technique that provides quantitative maps of tissue absorption and scattering properties based on the principles of diffuse optical spectroscopy.^22^^,^^23^^,^^25^ SFDI has been widely used in studies of normal skin,^26^^–^^28^ skin diseases,^29^^–^^31^ wound healing,^32^^,^^33^ reconstructive surgery,^34^^–^^36^ cancer,^37^^–^^41^ kidney transplant,^42^ and brain hemodynamics.^43^^–^^47^ SFDI can assess collagen denaturation (via changes in reduced scattering coefficient), hemodynamics, vascular damage, and edema (via absorption coefficient), making it a promising tool for developing objective burn severity metrics. Our group previously demonstrated that changes in the reduced scattering coefficient ($\mu_{s}^{\prime }$) and decreased tissue oxygenation in burn tissue regions, as measured with SFDI, correlate with collagen denaturation in the dermis and damage to the vascular network.^48^^–^^50^

Recently, we investigated the use of ML with calibrated reflectance ($R_{d}$) at multiple wavelengths and spatial frequencies to predict burn wound severity.^24^^,^^51^^,^^52^ In these studies, $R_{d}$ offers the benefits of a combined contrast between absorption and scattering properties. Our previous proof-of-concept work utilized a cubic support vector machine, a supervised algorithm, with SFDI inputs to produce maps of healing outcomes.^24^ We chose healing outcome as a binary classification of burn severity that is determined on day 28 postburn, where tissue was assessed to either heal with minimal contraction and scarring (associated with superficial or SPT burn) or not heal satisfactorily and require surgical intervention such as debridement and grafting (associated with DPT or FT burn).^24^ Our previous studies demonstrate that a 28-day period provides sufficient time for deeper burn wounds with healing potential to undergo re-epithelialization, allowing healing outcomes to be correlated with burn severity observed at earlier timepoints.^24^^,^^32^^,^^33^

Although classification based on healing outcome serves as useful proof of principle, it only indirectly reflects burn depth, which remains the standard metric in clinical assessment of burn severity. In this paper, we revisit the use of collagen denaturation depth (CDD) as a metric for reporting burn depth, as collagen denaturation is a hallmark of thermal damage in skin.^53^^–^^55^ Specifically, we focus on completely denatured collagen assessed using histology with birefringent dye Picrosirius red (PSR).^56^^–^^58^ We employed a two-step regression framework to derive a simple and interpretable relationship between SFDI $R_{d}$ and CDD while also relating CDD to our previously chosen burn severity metric, day-28 healing outcome. First, we used multiple linear regression (MLR) to generate a predictive model of CDD as a function of $R_{d}$ as measured with SFDI within 24 h after burn creation (which we refer to as day 1). Second, we used logistic regression (LogR) to develop a probabilistic and classification model of day-28 healing outcome using day-1 histology-derived CDD. We ultimately achieve a two-step regression model enabling SFDI measurements acquired within the first 24 h post-burn to predict CDD for that time point and day-28 healing outcomes as direct and indirect metrics of burn severity, respectively.

## 2 Approach Overview

**Fig. 1:**
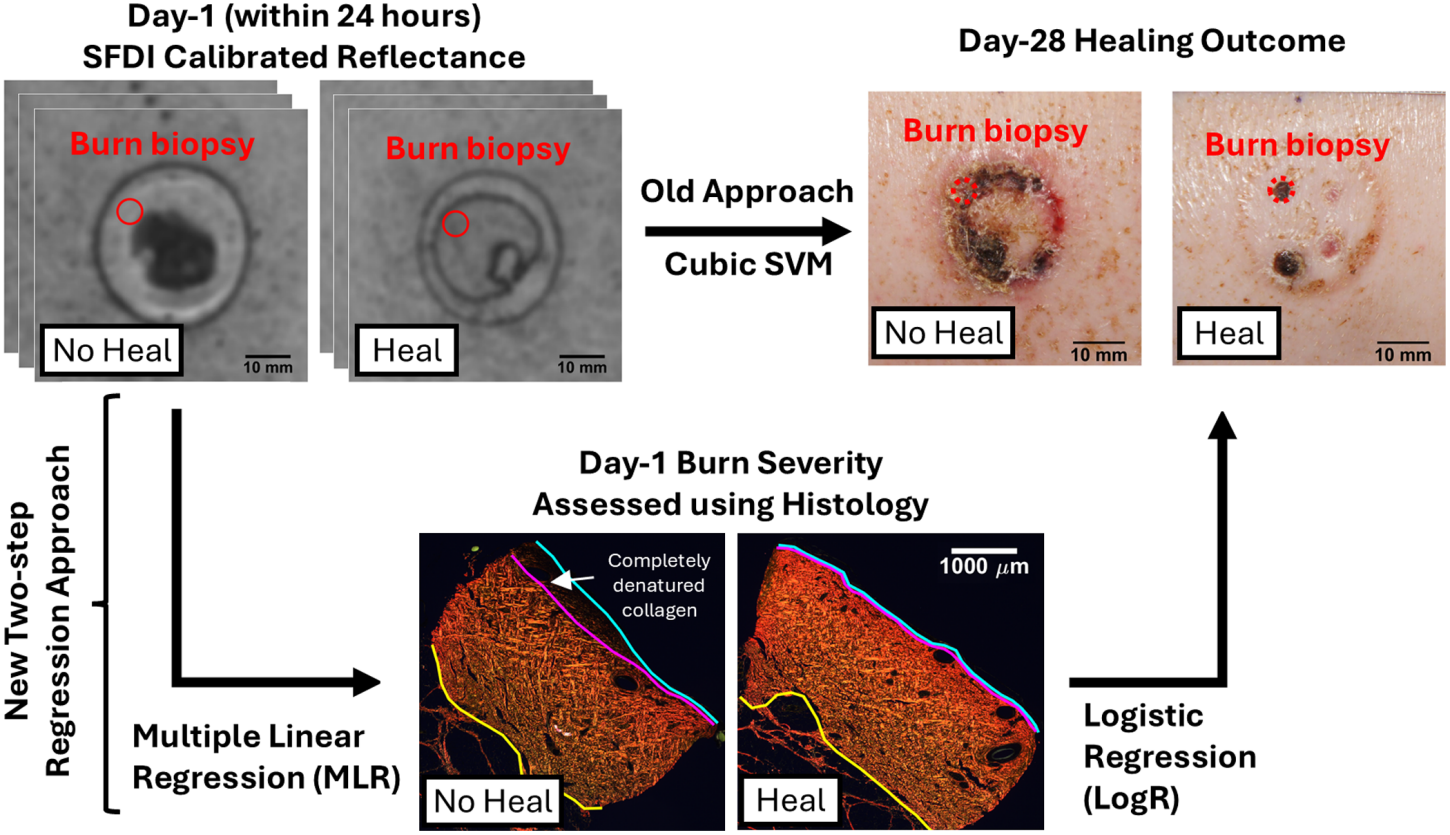
Graphical summary showing the employment of a novel two-step regression analysis approach to assess histology-derived burn severity as early as 24 h after burn creation (what we refer to as day 1) using SFDI calibrated reflectance ($R_{d}$), and how such a severity metric can predict healing outcome on day 28. The chosen histology-derived burn severity metric is completely denatured collagen, corresponding to dark pixels when stained with Picrosirius Red. Collagen denaturation is a hallmark of skin thermal injuries.

We implemented a two-step predictive framework (Fig. 1), integrating MLR and LogR to assess CDD as a direct metric of burn severity and healing outcomes.

Step 1: We used MLR to derive a predictive model of CDD using $R_{d}$ at the eight wavelengths and five spatial frequencies used previously.^24^ The MLR approach provides a continuous CDD response variable (units of micron) from $R_{d}$ images, differentiating this work from our prior classification approach.^24^
Step 2: We employed LogR to develop a probabilistic classification model for burn wound healing outcomes from histology-derived CDD. This step enables the study of histology-derived CDD as a predictor of burn wound healing.

## 3 Materials and Methods

### 3.1 Experimental Design

#### 3.1.1 Study timeline

We induced burn injuries of graded severity in a porcine model using an approach that we previously established.^24^ Biopsies were collected on days 1, 7, 14, 21, and 28 to assess thermal damage from histology. SFDI and conventional digital color photography were conducted before and after burn induction on day 0 and before biopsies on days 1, 3, 7, 14, 21, and 28. We performed histological analysis of the biopsied tissue using three stains: hematoxylin and eosin (H&E), Masson’s trichrome, and Picrosirius red. We previously used H&E and Masson’s trichrome to assess tissue damage and collagen denaturation, respectively.^24^^,^^50^^,^^59^ However, H&E and Masson’s trichrome stains may be difficult to interpret in a consensus fashion.^60^ In this study, we employed PSR, a known birefringent dye for collagen fibers.^54^^,^^56^ We imaged PSR-stained histology samples with polarization microscopy to assess collagen denaturation. Polarization microscopy of PSR-stained samples provides high-contrast images of completely denatured collagen, allowing for straightforward quantification of CDD. Table 1 lists all experimental procedures. All analyses presented were based on SFDI and histological data obtained at the 24-h postburn time point (which we refer to as day 1).

**Table 1:** Experimental timeline and procedures performed in chronological order on each day.

Experiment timeline	Procedures (in chronological order)
Day 0	Preburn SFDI & conventional photography
	Burn creation
	Immediate postburn SFDI & conventional photography
Day 1, 7, 14, 21	Prebiopsy SFDI
	Prebiopsy conventional photography
	Biopsy
	Postbiopsy conventional photography
Day 3	SFDI and conventional photography
Day 28	Prebiopsy SFDI & conventional photography
	Healing outcome determination using conventional photography
	Biopsy
	Postbiopsy conventional photography
	Euthanasia

#### 3.1.2 Animal handling and burn creation procedures

All animal experiments in this study adhered to a protocol (AUP‐24‐056) approved by the Institutional Animal Care & Use Committee at the University of California, Irvine. Four hybrid Yorkshire-Landrace pigs (two males, two females—labeled as P1, P2, P3, and P4) were employed. Each experiment lasted 28 days, and the study was performed over 34 months, with timing modulated in part by shared access to UCI’s large animal facilities. Animal weights and sex were recorded prior to the experiments and listed in Sec. 8 in Appendix B—Table 4.

**Fig. 2:**
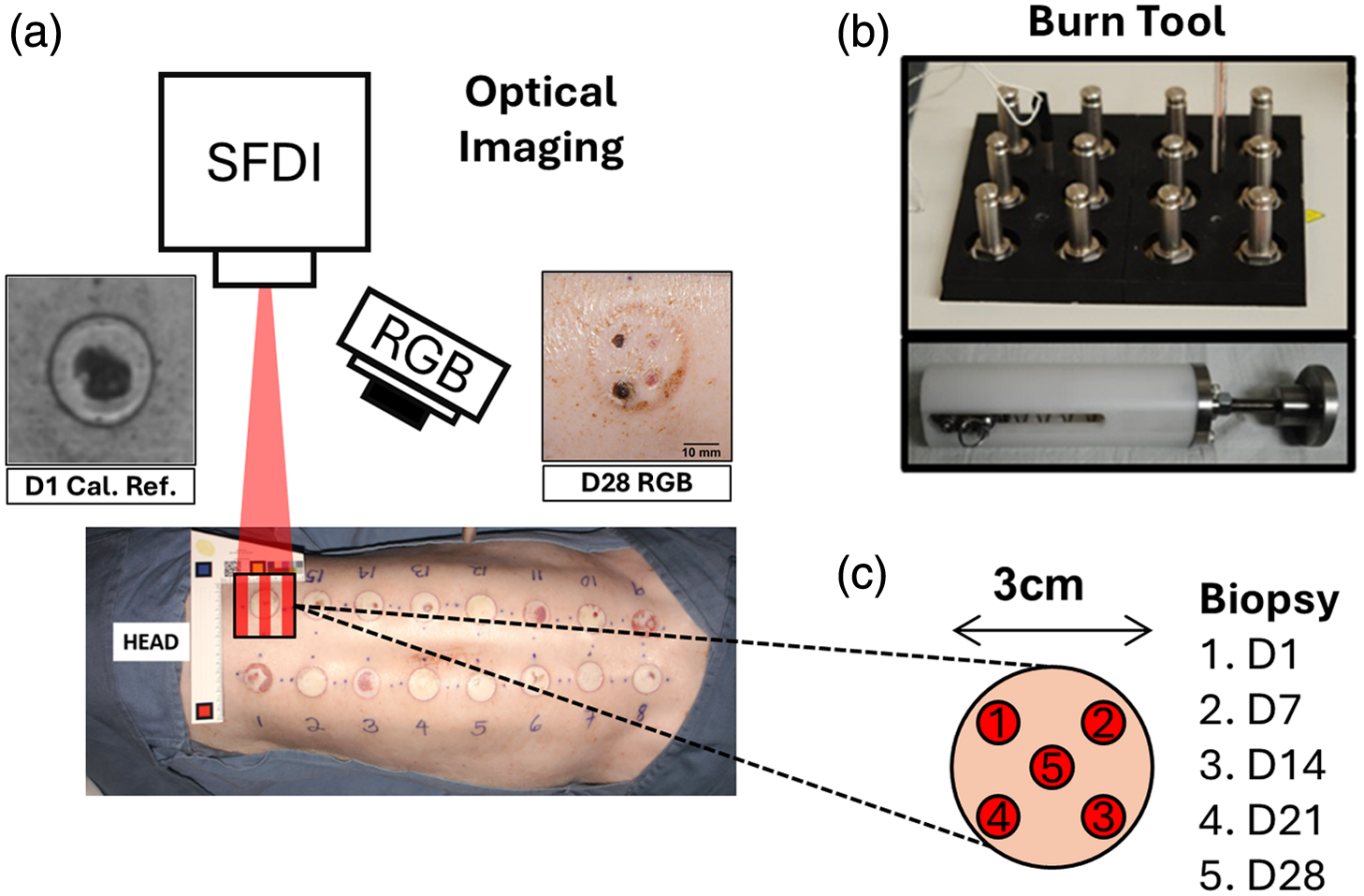
Experimental procedures. (a) SFDI and conventional color photography (RGB) were conducted on burns of varying severity, determined by contact durations ranging from 5 to 40 s, following our previous studies.^24^^,^^50^ (b) Representative images depict the burn tool, heating bath, and spring-loaded handle. (c) A schematic illustrates biopsies performed at different time points throughout the experiment.

The skin regions of interest were shaved and sterilized before creating controlled burn regions. The burn device, used in a previous set of studies,^24^^,^^49^ consisted of a 3- cm diameter brass rod heated to 100°C in an electronically controlled dry bath and mounted in a spring-loaded handle to ensure consistent pressure for each created burn [Fig. 2(b)]. Consistent with our previous studies, the device was applied to the skin for durations of 5, 10, 15, 20, 25, 30, 35, and 40 s along the dorsum on each side of the spine [Fig. 2(a)]. SFDI and conventional color photography were conducted immediately before and after burn creation on days 0, 1, 3, 7, 14, 21, and 28. SFDI imaging was performed before biopsies, while conventional photography was repeated both before and after biopsy. Further details regarding the anesthesia protocol and wound care can be found in Appendix B—Sec. 8.

#### 3.1.3 Spatial frequency domain imaging acquisition

SFDI measurements were conducted on days 0, 1, 3, 7, 14, 21, and 28 using five spatial frequencies (0, 0.05, 0.1, 0.15, and $0.2\;{\mathrm{mm}}^{-1}$) at eight wavelengths (471, 526, 591, 621, 659, 691, 731, and 851 nm) using a research-grade SFDI system (OxImager RS™, Modulim, Irvine, California, United States). As previously stated in our other porcine studies, we acquired three sets (i.e., repetitions) of data for each burn at each imaging session to ensure that we obtained at least one high-quality set of data per burn. We evaluated the quality of $R_{d}$ images to select one repetition that was least affected by motion and demodulation artifacts. In total, there are 40 $R_{d}$ maps, corresponding to eight wavelengths at five spatial frequencies, per 3 cm diameter circular burn region.

#### 3.1.4 Biopsy procedure, histology preparation, and imaging

On days 1, 7, 14, 21, and 28 post-burn creation, 6-mm punch biopsies were taken from each burn, which is consistent with the technique that we employed previously^24^ [Fig. 2(c)]. Despite efforts to apply uniform pressure to the skin with the heated brass rods, burns appeared heterogeneous. We previously reported on this well-known issue, which has been observed by other research groups, involving the creation of contact burns on porcine models.^49^^,^^50^^,^^61^^–^^63^ We harvested biopsies from tissue areas with visually homogeneous surfaces. Such regions were chosen away from the center of the burn, in the order of sampling date listed in Fig. 1(c).

In this study, we focused on CDD as the sole metric of burn severity. We chose this metric for two reasons: 1) CDD historically corresponds with the depth of thermal injury^53^^,^^64^^,^^65^ and 2) CDD can be objectively quantified using PSR and polarization microscopy as outlined below^56^^–^^58^ while traditional histological grading of true burn damage depth is prone to inter- and intra-personnel discrepancies.^60^^,^^66^ The main cause of these discrepancies is the lack of consensus on histological parameters (e.g., coagulated blood vessels, collagen denaturation, and immune cells infiltration) used in the grading process. Even with a rubric of specific histological parameters, the large number of features needed for the analysis adds to intra- and inter-personnel variation.^60^

**Fig. 4:**
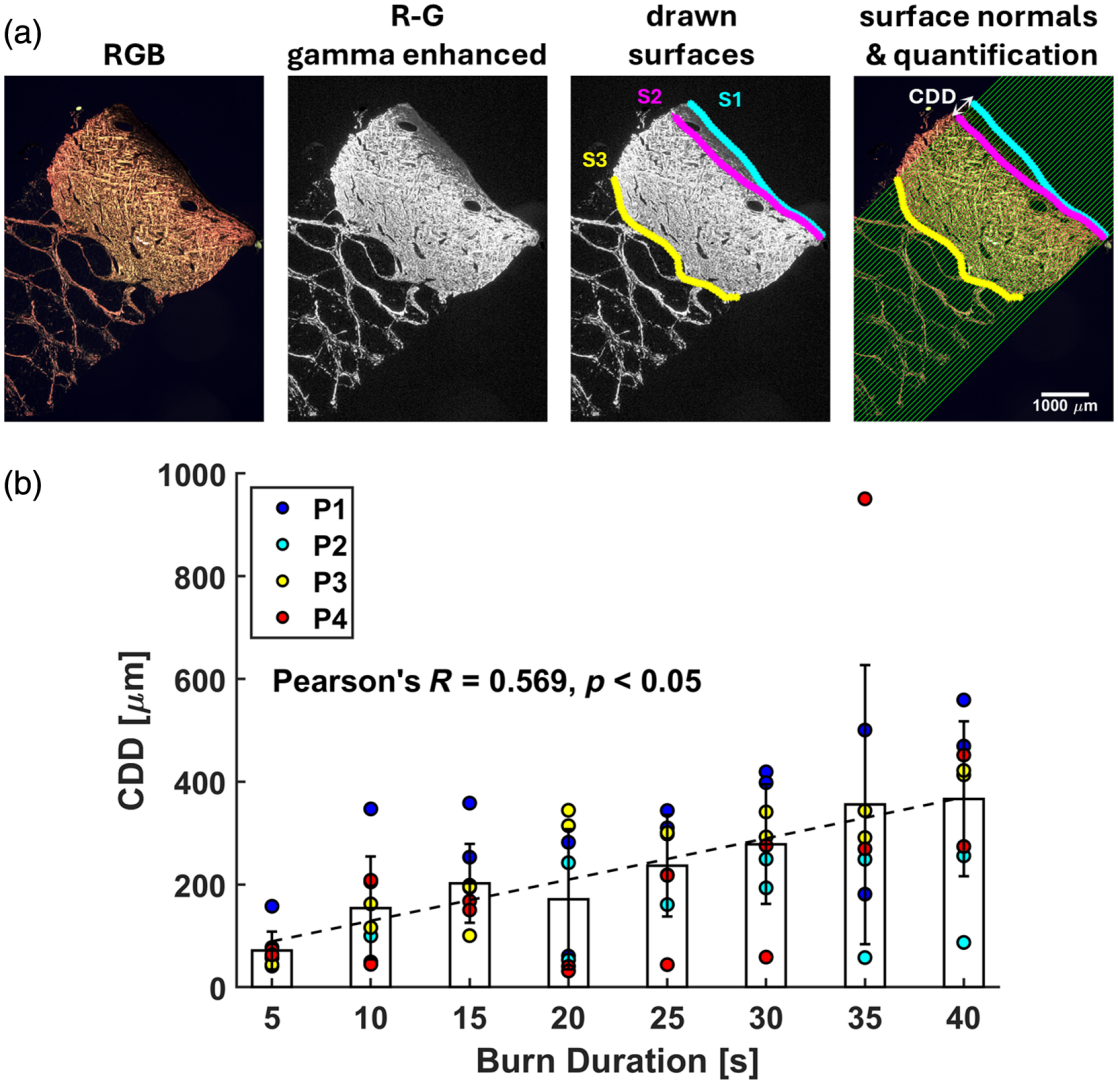
Histology analysis workflow and results. (a) Representative images illustrate the histology processing pipeline, highlighting the dermis surface (S1, cyan), extent of denatured collagen (S2, magenta), dermis base (S3, yellow), and surface normal (green lines) used to calculate collagen denaturation depth (CDD). (b) The distribution of CDD across all four porcine subjects (P1, P2, P3, and P4) demonstrates a consistent increase with longer burn durations. The bar graphs and associated error bars represent the mean and standard deviation of CDD measurements for each burn duration group. Pearson’s correlation analysis was performed with a significance threshold of p=0.05.

Histology slides of the punch biopsies were stained with PSR (ab150681, Abcam, Cambridge, United Kingdom), a stain that enhances the birefringent properties of healthy collagen fibers. Cross-polarization microscopy (0 deg and 90 deg) was performed using a Jigtech polarization package by Daitron (N11599-00R-002, Keyence, Osaka, Osaka, Japan) and a Keyence BZX microscope (Keyence, Osaka, Osaka, Japan) at 2x magnification with fixed exposure time. The captured images enabled us to distinguish complete denaturation (i.e., low intensity or dark pixels) from healthy and partially damaged collagen (i.e., high intensity or bright red pixels) [See Fig. 4(a) in Sec. 3.2.3]. This methodology was previously outlined by Campbell et al.,^57^ which is detailed in Appendix B—Sec. 8. CDD can then be digitally quantified as discussed in Sec. 3.3.

#### 3.1.5 Color (RGB) photography

We used a commercial camera (NEX-3, SONY, Tokyo, Japan) for conventional color photography, as was done in our previous studies. Color images were taken before and after burn creation on day 0 and before and after biopsy on days 1, 3, 7, 14, 21, and 28.

### 3.2 Data Sampling and Preparation

#### 3.2.1 SFDI processing and sampling

SFDI $R_{d}$ images were generated following the methodology described by Cuccia et al.^22^ $R_{d}$ images were stored as an image cube (image width × image height × feature index), where the feature index corresponds to calibrated reflectance values at various spatial frequencies and wavelengths. Section 8 in Appendix B—Table 5 displays the full feature list with the corresponding wavelengths and spatial frequencies.

**Fig. 3:**
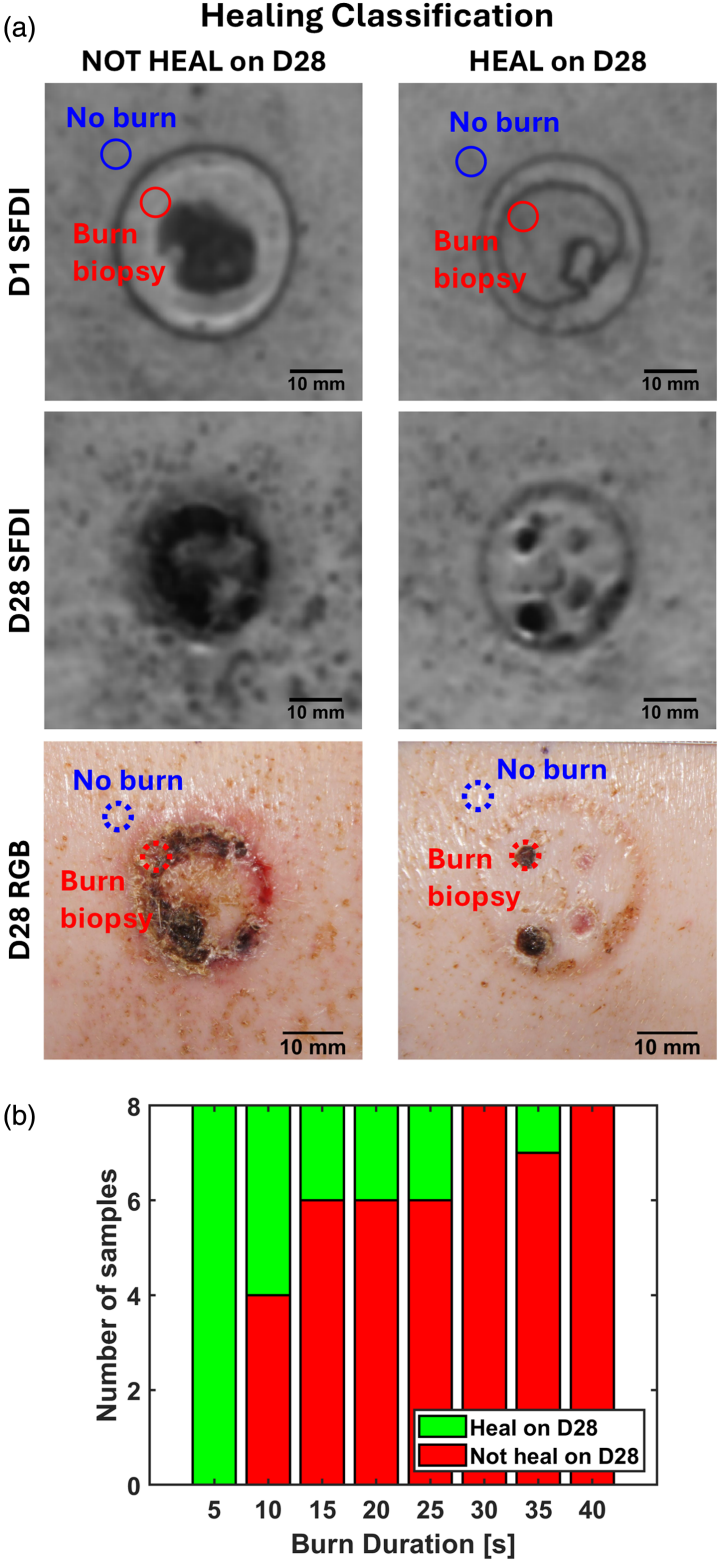
Sampling procedure of SFDI data. (a) Representative $R_{d}$ images of chosen ROIs (red circles—burn, blue circles—no burn) for burns that did not heal (left) and that did heal (right) by day 28 (D28). (b) The distribution of healed samples across burn durations demonstrates an expected decline in healing with longer burn durations.

From the day-1 SFDI $R_{d}$ image cube, we cropped a 221×221-pixel (or 6×6  cm) subregion centered around the burn, which we defined as the full field-of-view (FOV) images in this manuscript. We applied a circular region of interest (ROI) with a 10-pixel (∼2.73  mm) radius to sample data from the biopsy site within the burn [Fig. 3(a)]. This ROI size was selected to approximate the biopsy dimensions. The ROI location was determined using post-biopsy conventional color images [Fig. 3(a)—red circles]. We employed manual visual registration between SFDI and color images to select the ROIs due to wound healing and contraction between day 1 and day 28, which hinders any automatic registration efforts. In addition, an identical ROI shape and size were used to sample unburned skin regions near the biopsy site [Fig. 3(a)—blue circles].

#### 3.2.2 Determination of healing outcome on day 28

The healing outcome of the biopsied area was assessed through visual examination of color photographs taken on day 28 by experienced burn researchers, including Drs. Joe and Kennedy. Specifically, we evaluated the tissue surrounding the selected ROI to determine whether it had healed with acceptable cosmesis by day 28. Figure 3 shows the binary classification system that was employed: “Heal” for tissue that healed on day 28 without significant scarring and contraction (suggesting that the burn was superficial or SPT), and “Not Heal” for tissues that demonstrated eschar or significant scarring and contraction on day 28 (suggesting that the burn was DPT or FT that would have been referred to surgery in a clinical setting). This binary healing outcome classification corresponds with the clinical decision of performing grafting for severe burns.^24^ As the biopsy was originally taken from a visually homogeneous sub-region on day 1, we assume that the healing outcome of the surrounding tissue reflected the condition of the biopsy ROI, as previously done in Ref. 24. Figure 3(b) illustrates the distribution of healing outcomes across various burn durations.

#### 3.2.3 Histology processing

We developed a histology processing approach to assess CDD in a semi-automated manner. Here, we reiterate that we defined complete denatured collagen depth as CDD, which is inherently shallower than altered or damaged collagen depth in surface thermal injuries. First, we observed that completely denatured collagen remains dark in color images when all three color channels (i.e., R, G, B) are shown [Fig. 4(a)]. Conversely, healthy or incompletely denatured collagen remains bright red [Fig. 4(a)]. From the polarization microscopy image, we generated a differential image between the R and G channels to visualize the extent of CDD.^57^ Contrast between completely denatured and healthy collagen was enhanced using MATLAB’s “imadjust” function with a gamma value of 0.5. From the gamma-enhanced map, three lines were manually traced: the dermis surface (S1), the extent of denatured collagen (S2), and the dermis base (S3) [Fig. 4(a)]. A line was fit through S1 points, from which 50 surface-normal vectors were drawn. For surface-normal vectors intersecting all three lines S1-S3, the depth of completely denatured collagen was defined as the distance between the intersection with S1 and the intersection with S2. This approach, which automatically draws the surface-normal vectors from a fitted line along the manually drawn dermis surface, enables us to obtain CDD measurements across all histology samples. Measured from the dermis surface visible through PSR staining, a positive CDD indicates damage beyond the epidermis, consistent with SPT and deeper burns.

### 3.3 Development of a Predictive Model for Collagen Denaturation Depth Using Calibrated Reflectance ($R_{d}$) and Multiple Linear Regression

We previously demonstrated that $R_{d}$ data taken on day 1 postburn could accurately classify burn wound healing outcome on day 28.^24^ Regions that healed without significant scarring or contraction correspond to burns having a severity of ST or SPT (i.e., burns that typically heal with acceptable cosmetic appearance under conditions of adequate nonsurgical wound care). Areas that presented eschars, significant scarring, and contraction correspond to burns that were DPT or FT (i.e., burns that should have been subject to surgical management).^67^

In this study, we developed an MLR model using day-1 $R_{d}$ data to evaluate same-day CDD. Because histology is difficult to precisely match to $R_{d}$ data at the individual pixel level, the median histology-derived CDD value of the 50 drawn surface normals was used to represent the entire ROI. The median value was chosen to mitigate the effects of any outlying measurements of CDD caused by processing artifacts of the histology samples, such as tissue tearing near the biopsy edge. We then utilized all 40 $R_{d}$ maps to develop the baseline general MLR model through the ordinary least squares (OLS) method. Performance was evaluated using leave-one-subject-out cross-validation (LOSO CV) with metrics such as root mean squared error (RMSE) and adjusted $R^{2}$.^68^ The model was then applied to every pixel in the $R_{d}$ image to create full field-of-view (FOV) predicted maps of CDD. Mathematical terminology, notations, and definitions are defined in Sec. 7 in Appendix A at the end of the paper.

### 3.4 Development of Probability and Classification Models of Healing Outcomes from Histological Collagen Denaturation Depth Using Logistic Regression

Our MLR model above predicts day-1 CDD with same-day $R_{d}$ as inputs; however, our original ML model outputs day-28 healing outcome classifications.^24^ As noted above, we selected CDD as a preliminary quantitative histology-derived surrogate for burn severity in this study and reiterate that the common burn severity metric is typically burn damage depth, which is assessed using multiple histological parameters.^2^^,^^60^^,^^66^^,^^69^ Here, we evaluated whether day-1 histology-derived CDD is an early indicator of healing outcomes observed on day 28. LogR was employed with day-1 histology-derived CDD as the predictor variable and day-28 healing outcomes as the response variable to create probabilistic and classification models as detailed below. Mathematical terminology, notations, and definitions are defined in Sec. 7 in Appendix A.

A simple LogR with a logit link function (defined in Sec. 7 in Appendix A) was performed with day-1 histology-derived CDD as predictor and day-28 healing outcome as response. LOSO CV was used to evaluate performance, and the area under the receiver operating characteristic (AUC ROC) curves was calculated for both training and testing sets. Subsequently, a general model was generated using data from all four animals.

To maximize sensitivity and specificity of the classification model, we used the maximum Youden J statistic and its corresponding histology-derived CDD value as a threshold for the general LogR model. The general probabilistic and classification models were applied to every pixel within the full-FOV MLR-predicted CDD map derived from the MLR.

## 4 Results

### 4.1 Day-1 Histology-Derived CDD and Day-28 Healing Outcome Track with Burn Durations

The clinical impression results showed that burns with shorter durations were more likely to heal satisfactorily by day 28 [Fig. 3(b)]. Furthermore, histology results demonstrated a significant moderate linear correlation between histology-derived CDD, as determined by PSR histology, and burn durations [Fig. 4(b), Pearson’s correlation coefficient R=0.569, p-value<0.05]. Specifically, 100% of samples exposed to 5 s of burn contact healed on day 28, whereas less than 50% of samples healed after burn contact times of 10 s or longer. Overall, healing outcomes exhibited a clear decreasing trend with increasing burn durations.

### 4.2 MLR Model Using all 40 $R_{d}$ Measurements Predicts CDD with Approximately 100 μm Accuracy

LOSO CV of the MLR model yielded a mean RMSE of 105  μm with a standard deviation of 47  μm across all folds. The mean adjusted $R^{2}$ for the linear fit was 0.71, with a standard deviation of 0.08. RMSE values ranged from 69 to 171  μm, whereas adjusted $R^{2}$ values ranged from 0.66 to 0.83. The general model achieved an adjusted R² of 0.69 for the linear fit, as summarized in Table 2.

**Table 2:** Summary of the performance metrics for each LOSO CV fold and the general model in the baseline MLR model utilizing all 40 $R_{d}$ features. All numerical values of RMSE are rounded to the nearest μm.

	Train data	Test data	RMSE (μm)	Adjusted $R^{2}$
LOSO CV Fold 1	P1,P2,P3	P4	171	0.83
LOSO CV Fold 2	P1,P2,P4	P3	69	0.66
LOSO CV Fold 3	P1,P3,P4	P2	72	0.70
LOSO CV Fold 4	P2,P3,P4	P1	111	0.68
General model	P1,P2,P3,P4			0.69

**Fig. 5:**
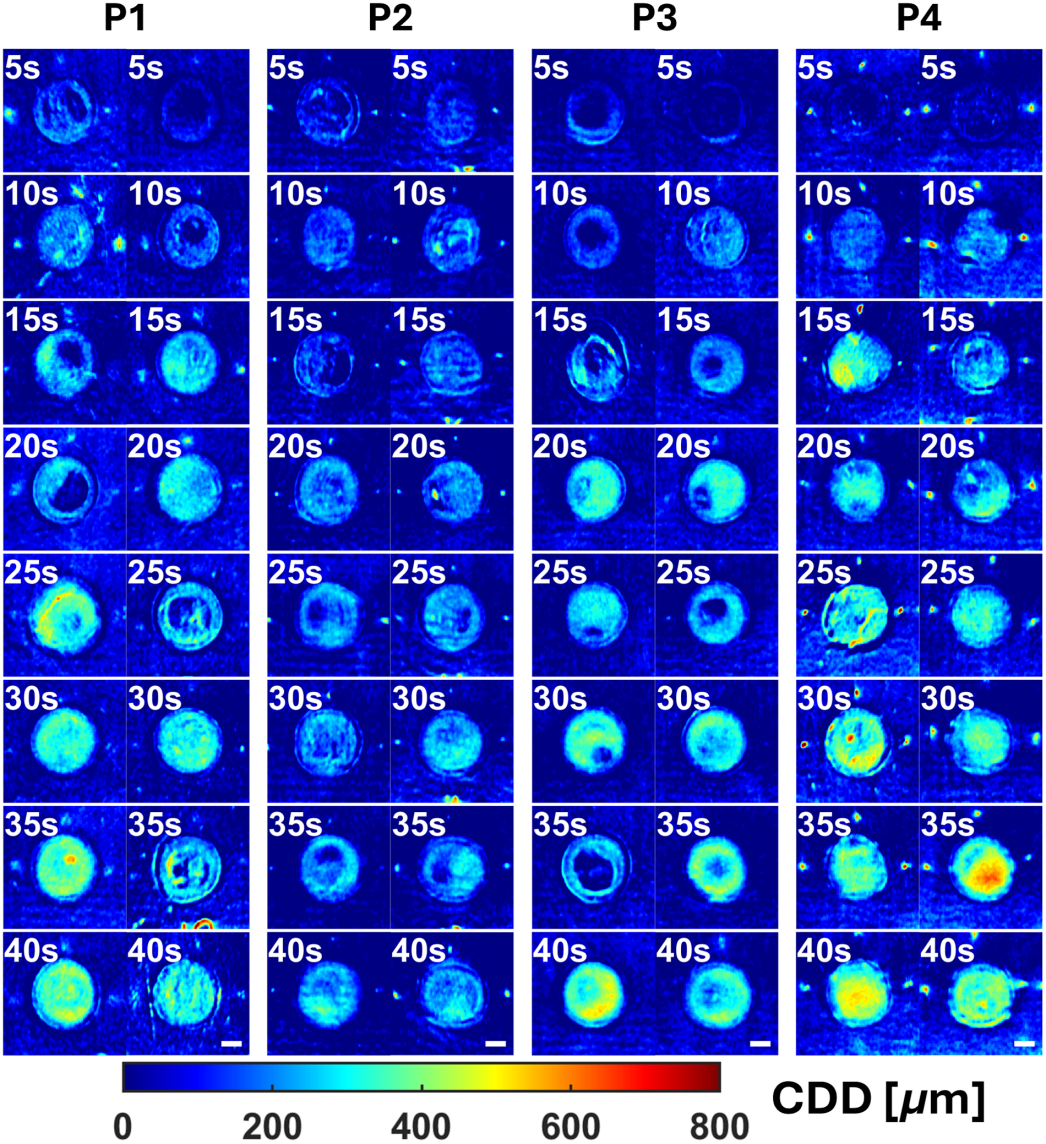
Spatial maps of CDD for pigs P1 through P4 across all experimented burn tool contact times (5 to 40 s). Application of the general model to individual pixels yields full-FOV maps of CDD. There exists a trend of increasing CDD with increasing burn time (top left corner) for P1, P3, and P4, but not as evidently in P2. Scale bars (white—bottom right) denote 10 mm.

The general model was then applied to every pixel to create a full field-of-view (FOV) map of predicted CDD. A visual trend of increasing CDD with longer burn durations (top-to-bottom) was observed for P1, P3, and P4, but not as clearly for P2 (Fig. 5). We discuss this deviation in the discussion section.

### 4.3 LogR Model Demonstrated Predictive Capabilities for Healing Outcomes

**Fig. 6:**
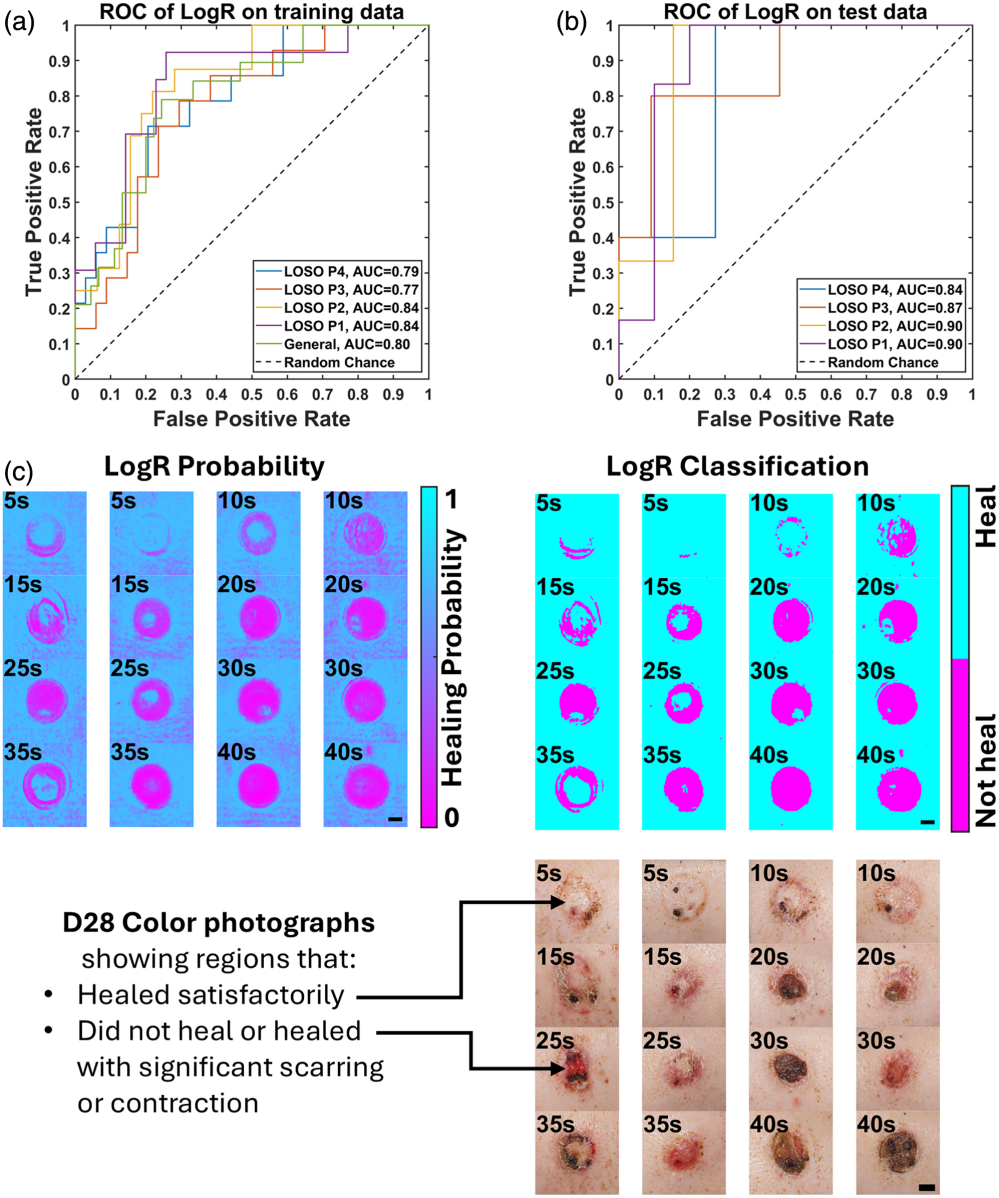
Logistic regression (LogR) results for predicting healing outcomes based on histology-derived CDD. (a) Receiver operating characteristic (ROC) curves for the training data across all LOSO CV folds and the general model, with calculated areas under the curve (AUC). (b) ROC curves for the testing data of all LOSO CV folds, accompanied by their respective AUC values. (c) Representative full-FOV healing probability and classification maps generated by applying the general LogR model to individual pixels, along with day-28 full-FOV color photograph showing the heterogeneous healing outcome of each burn. Scale bars (black—bottom right) denote 10 mm.

LogR was performed using day-1 histology-derived CDD as the predictor and day-28 healing outcomes as the response. ROC curves were associated with a mean AUC of 0.81 for the training data and 0.88 for the testing data across all folds of LOSO CV [Figs. 6(a) and 6(b)]. The general model achieved an AUC of 0.80, with a maximum Youden’s J statistic of 0.55, corresponding to a healing probability threshold of 0.31 and ∼181  μm in histology-derived CDD. All outputs for each subject are listed in Sec. 8 in Appendix B.

It is worth noting that a predictive model of day-28 healing outcome from day-1 SFDI and histology data suggests that histology-derived CDD is an early indicator of healing outcome. Applying the general LogR model to every pixel of the predicted CDD maps from MLR yielded full-FOV maps of healing probability, and application of the classification threshold yielded maps of the “Heal” and “Not Heal” regions [Fig. 6(c)]. Because the data were only trained with “Heal” and “Not Heal” classes, the surrounding unburnt skin region was also considered as “Heal”. Overall, the healing classification map visually agrees with color photographs taken on day 28 [Fig. 6(c)].

## 5 Discussion

In this study, we successfully demonstrated that SFDI can predict burn-induced collagen denaturation and healing outcomes through a novel two-step regression framework. Our approach achieved clinically relevant accuracy with a mean RMSE of 105  μm for CDD prediction (adjusted $R^{2}=0.71$) and an ROC AUC of 0.88 for healing outcome classification. These results establish important quantitative relationships among post-burn day-1 SFDI-calibrated reflectance ($R_{d}$), day-1 histology-derived CDD, and day-28 healing outcomes. In step 1, we employed MLR to develop a predictive model for day-1 CDD based on same-day SFDI $R_{d}$ data. In Step 2, LogR was employed to predict day-28 healing outcome using day-1 histology-derived CDD results.

When applying the general MLR and LogR models to the full-FOV images, we observed an expected general trend of increasing CDD and decreasing healing probabilities for burns with longer contact times. LogR analysis determined a healing threshold for CDD at 181  μm. We reiterate that our CDD measurements correspond with the completely denatured collagen depth, which is often shallower than the damaged collagen depth and burn depth.^50^^,^^53^^,^^60^

### 5.1 Practicality of Burn Wound Severity Assessment and Debridement Guidance Potential

In clinical practice, severe burn management often requires the removal of nonviable tissue to prevent infection and promote recovery.^70^^–^^73^ Surgeons typically debride until the first signs of bleeding occur, signaling the presence of viable dermis. Hence, estimating burn damage depth is implicit for guiding effective debridement. Although histopathology is regarded as the “gold standard” for assessing burn damage depth, the literature suggests that pathologists struggle with consensus when multiple experts are involved.^60^^,^^66^ Traditional grading of burn histology also assesses altered or partially damaged collagen with stains such as Masson’s trichrome or H&E. Assessing altered collagen involves identifying features such as discoloration and the overall shape or architecture (e.g., appearing thin, flattened, or fissuring), which is often up to interpretation and thus subjective.^60^^,^^66^^,^^69^ Furthermore, traditional burn histology grading also assesses vascular blockage and damage to dermal appendages such as hair follicles.^60^^,^^66^ In fact, burn damage often exceeds the depth of completely denatured collagen.^69^ Therefore, our MLR model, which outputs CDD, provides a minimum required depth for debridement guidance. Future studies can substitute CDD with other damage depth metrics (e.g., cell damage and blood vessel coagulation) and utilize a similar two-step regression framework to derive a predictive model.

### 5.2 Potential Explanations for Subject P2’s Modest Increase in CDD with Longer Burn Durations

**Fig. 7:**
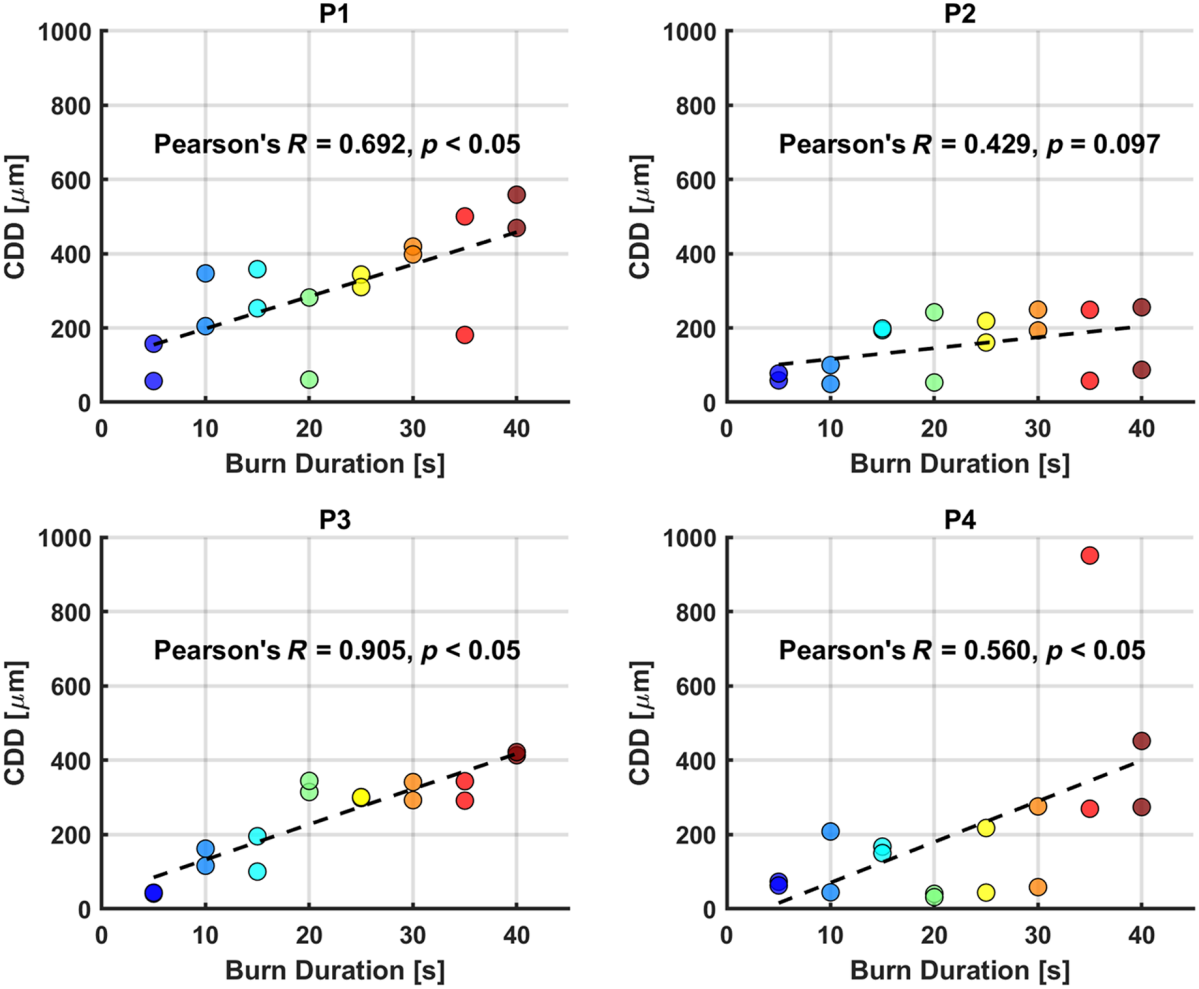
Individual correlation analysis of CDD and burn duration reveals significant positive Pearson’s correlations for porcine burn subjects P1, P3, and P4, whereas no significant correlation is observed for subject P2. Colors (blue to brown) were used to indicate burn durations.

Although our overall model demonstrated strong predictive capability, individual subject variability provided important insights into model robustness and potential sources of error. Notably, subject P2 showed a modest increase in CDD with longer burn durations (Fig. 5), diverging from the clear increasing trend observed in subjects P1, P3, and P4. This divergence from the increasing trend observed in subjects P1, P3, and P4 was attributed to P2’s histology results, which showed a weaker correlation to burn duration as illustrated in Fig. 7. Independently, P2 did not show a significant linear correlation between CDD and burn duration (Pearson’s correlation coefficient R=0.429, p-value=0.097). Our animal welfare records did not indicate health-related issues for P2 that could explain this insignificant trend. Thus, potential reasons for this insignificant trend for P2 include the burn tool not being properly heated to 100°C prior to burn creation and suboptimal contact between the tool and the skin surface. Conversely, subjects P1, P3, and P4 demonstrated significant correlations, with p-values below 0.05 and Pearson’s R values of 0.692, 0.905, and 0.560, respectively (Fig. 7).

### 5.3 Performance Comparison with Other Machine Learning Approaches

Our two-step regression approach represents a novel application of SFDI for burn severity assessment, offering several advantages over existing methods. To place our results in context, we compare our performance with other ML approaches for burn assessment, noting that this study represents the first attempt to assess completely denatured collagen depth using SFDI. Previously, Wang et al. utilized HSI and rotational feature subspace ensemble regression with K-nearest neighbor (RSER-KNN) to predict burn depth determined by H&E histology.^14^ Similar to our experimental design, the group used a brass rod heated to 100°C to induce burns on a pig’s torso. The HSI approach utilized a short-wave infrared (SWIR) spectrum between 950 and 1650 nm with 10-nm steps. In this spectrum, the source of contrast is mostly water from edema and damaged fat for FT burns.^74^^,^^75^ Using a sample size of one animal used for both training and testing data, the model achieved a mean relative error of 7% for a range of burn depths between 127.36 and 5293.33  μm. This corresponds to an average absolute error of 32  μm for the burn durations comparable to ours (i.e., 5s to 40s). Relative to our MLR model, Wang’s nonlinear RSER-KNN demonstrates improved model fit and predictive robustness. Nonetheless, both approaches require a larger sample size to adequately assess and validate their generalizability across broader populations.

Beyond depth prediction, our framework also addresses the critical clinical need for early healing outcome prediction. Our LogR model’s performance in this binary classification task compares favorably with other imaging-based approaches. Specifically, the general model enabled the prediction of day-28 healing outcome based on CDD maps obtained from day 1 with an AUC of 0.80. The current model only yields two classifications of “Heal” or “Not Heal” on day 28. The maximum Youden’s J statistic was 0.55, corresponding to a true positive rate (i.e., sensitivity) of 0.79 and a true negative rate (i.e., specificity) of 0.76. Our previous SVM model utilized SFDI $R_{d}$ to separate imaged tissue into four classifications: unburned skin, hyper-perfused periphery, burned regions that, with conservative wound care, would heal by day 28, and regions that would not heal by day 28 that would require excision and grafting.^24^ The performance was calculated as a confusion matrix (precision and recall) for each class and is not directly comparable to the current binary classification model. The overall accuracy of the current LogR binary classification model is 75%, whereas the overall accuracy of the previous multiclass SVM model is 92.5%. However, the previous model was limited to the same animals used for training and testing.

Historically, several imaging modalities have been investigated to assess burn wound severity. The recent advancement of ML algorithms has enabled the development of classification models for burn severity assessment.^76^ Several groups also demonstrated the efficacy of color photography in clinical burn depth classification, with a mean of 80.9% accuracy without deep learning and 86.2% with deep learning.^76^^–^^80^ Schulz et al. applied LogR to a self-defined burn index obtained from HSI to derive a probabilistic model of day-21 naturally healing versus nonhealing and observed an AUC of 0.81 at 24 h, 0.66 at 48 h, and 0.81 at 72 h postburn.^81^ Thatcher et al. utilized MSI with a convolutional neural network to classify clinical burn depth impressions with a sensitivity of 0.81 and a positive predictive value of 0.97.^15^ Although our general LogR model demonstrates comparable performance within 24 h postburn (AUC = 0.80, sensitivity = 0.79, specificity = 0.76, and accuracy = 75%), it is important to note that our classification is based solely on the binary healing outcome at day 28, rather than the clinically standard burn depth assessment.

**Fig. 8:**
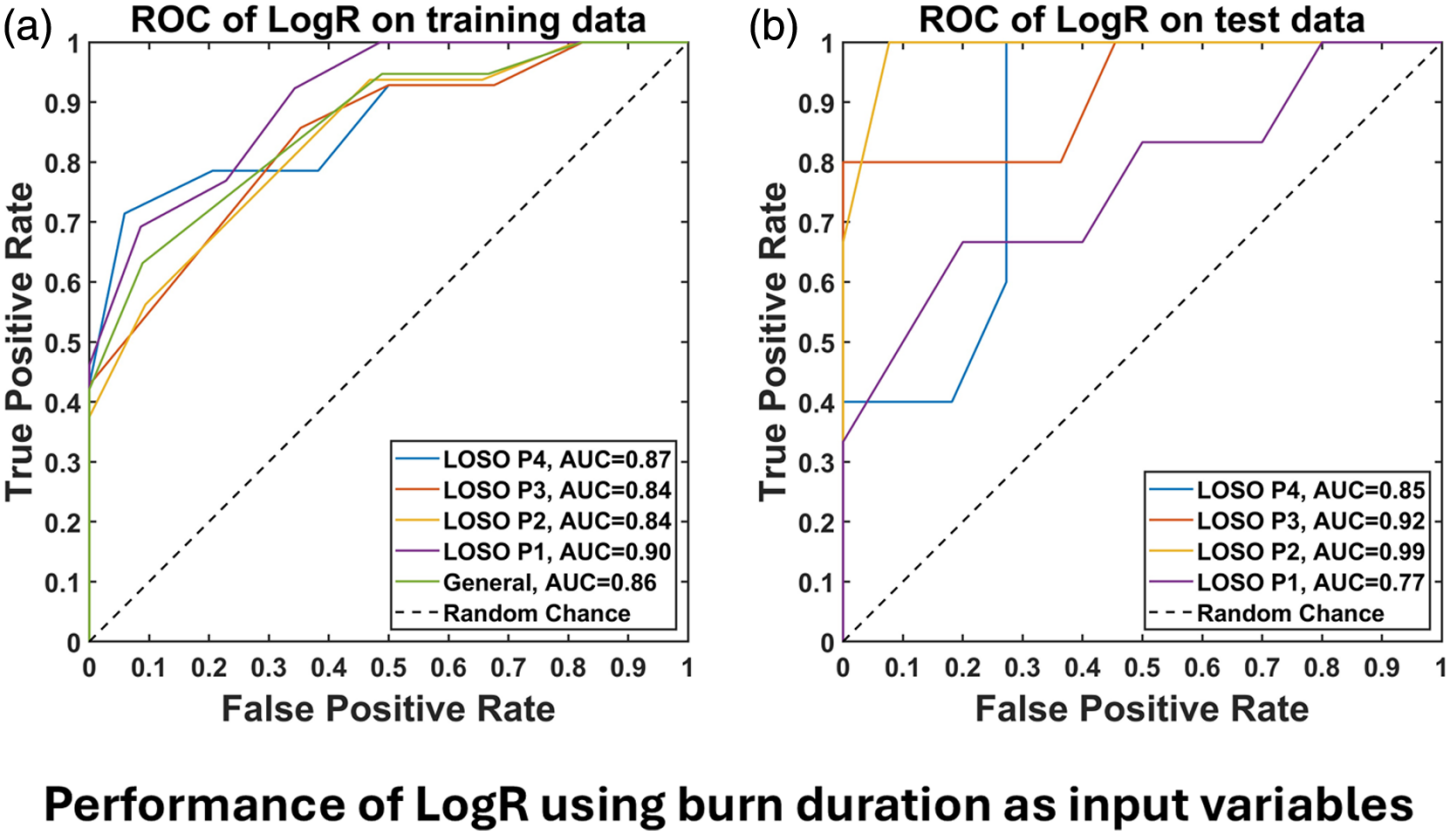
Logistic regression (LogR) results for predicting healing outcomes based on burn duration show similar performance to the CDD-based model. (a) Receiver operating characteristic (ROC) curves for the training data across all LOSO CV folds and the general model, with calculated areas under the curve (AUC). (b) ROC curves for the testing data of all LOSO CV folds, accompanied by their respective AUC values.

We also evaluated a LogR model using burn duration as input and compared it to the CDD-based model (Fig. 8). Both models showed comparable performance, with AUCs of ∼0.8 to 0.9 for training data and ∼0.7 to 0.9 for test data under LOSO cross-validation. This similarity reflects the correlation between CDD and burn duration [Fig. 4(b)] and the observed decline in healing probability with longer burn times [Fig. 3(b)]. This finding indicates that burn duration may serve as a naïve predictor of healing. However, its clinical utility is limited due to the impracticality of accurately determining burn contact time, initial temperature, and pressure applied.

### 5.4 Preliminary Interpretation of Important SFDI Features in MLR Model

In this preliminary attempt to identify important SFDI features for burn severity assessment, we interpreted the regression coefficients (i.e., β) of the general MLR model as the feature’s contribution rank, given that the feature’s p-value of t-statistic showed significance (p<0.05). Specifically, the high absolute value of a regression coefficient suggests that the feature is crucial to the model. The 10 most important SFDI features were identified based on the absolute regression coefficients (β) from a general MLR model trained on data from four animals. These features span wavelengths from green (526 nm) to orange/red (591 to 731 nm) and include spatial frequencies ranging from 0 to $0.2\;{\mathrm{mm}}^{-1}$ (Table 3).

**Table 3:** List of the 10 most important SFDI features (with p-values for t-statistics <0.05) as determined by the regression coefficients of the general MLR model.

Feature number	Center Wavelength [nm]	Spatial frequency $\left[{\mathrm{mm}}^{-1}\right]$
16	621	0
9	526	0.15
6	526	0
19	621	0.15
11	591	0
15	591	0.2
28	691	0.1
12	591	0.05
27	691	0.05
35	731	0.2

In SFDI, low spatial frequencies are more sensitive to absorption, whereas high spatial frequencies emphasize scattering effects.^22^^,^^82^ The presence of high spatial frequencies among key features suggests that scattering changes are associated with burn severity, potentially due to collagen denaturation. This is consistent with our group’s early work relating burn severity to reduced scattering coefficient.^50^ In addition, increasing spatial frequency also corresponds with decreasing effective probing depth.^22^ Notably, the first six features include 526, 621, and 691 nm at both planar ($0\;{\mathrm{mm}}^{-1}$) and high (0.15 to $0.2\;{\mathrm{mm}}^{-1}$) spatial frequencies, indicating absorption differences, possibly from blood coagulation,^83^ between superficial and deeper tissue layers as a source of contrast for burn severity. This association may be explained by CDD’s strong correlation with burn depth, which also correlates with vascular damage.^60^^,^^66^ All features and their associated regression coefficients are listed in Table 5 in Appendix B in Sec. 8.

It should be noted that this is only a preliminary analysis. Robust feature selection requires a more thorough approach that addresses the disproportionate distributions existing in diffuse reflectance data (i.e., high spatial frequencies tend to have much lower diffuse reflectance values, which can inflate the regression coefficients). Our follow-up work focuses on fielding such approaches, which can include standardization,^68^ regularization,^84^ information theory,^85^ and bootstrapping.^68^

### 5.5 Study Limitations

Preclinical burn models are well-documented in the literature to yield heterogeneous wounds.^2^^,^^69^^,^^86^^,^^87^ Here, we excised tissue from visually homogeneous regions during biopsy sampling and used the median CDD value from graded histology within the ROIs in the MLR analysis. However, this process may introduce larger errors in MLR modeling where a single CDD value is not representative of the spatial expanse of the heterogeneous wound and does not account for potential variation in staining or tissue dehydration among different samples. Furthermore, approximating the spatial alignment of ROIs from day 1 to day 28 was challenging due to burn contraction and progression over time.^2^ Biopsy sampling also disturbed the tissue and altered the wound dynamics and progression.

Another limitation of our study is that all burns were induced on the dorsal region of the porcine subject, where the skin is relatively thick.^88^^,^^89^ For example, at 3 to 4 months old, porcine dorsal skin thickness ranges from 1200 to 1600  μm, depending on the relative position to the head, which is significantly thicker than the belly skin thickness, which often ranges from 1000 to 1100  μm.^88^^,^^89^ As a result, the absolute measurements of denatured collagen depth may not accurately reflect the extent of tissue damage that would occur in other thinner-skinned areas. Furthermore, porcine skin is generally thicker than human skin of the same relative age.^90^^,^^91^ Thus, using a relative measurement of CDD (e.g., %CDD of the dermal layer) may offer greater versatility and translatability. However, to our knowledge, such a metric is not currently supported or validated in existing literature as an indicator of skin thermal damage.

Lastly, the application of the MLR model to human studies is constrained by the frequent unavailability of biopsy consent. To address this limitation, recent clinical burn studies have established “truthing panels” composed of multiple burn experts who collaboratively assess injury severity.^15^^,^^92^ These studies leverage biopsies collected during operative procedures, where tissue is already being excised through debridement or resection. Access to such datasets, when available, would facilitate evaluation of the current proposed approach’s (i.e., MLR + LogR) performance.

### 5.6 Future Work

In this study, we fielded only CDD as a standalone histology-derived burn severity metric to be used in our MLR and LogR analysis pipeline. However, other histopathological metrics, such as the number and existence of blood vessel coagulation, hair follicle damage, and the extent of inflammatory cell infiltration, may also aid in the determination of burn severity and healing outcome.^60^^,^^66^^,^^69^ Future studies may incorporate these other histopathological metrics into a multivariate regression analysis (e.g., multivariate linear regression and multivariate LogR) to improve model performance. Furthermore, employment of nonlinear methods (e.g., neural networks, random forest, and support vector regression) may enhance the performance of the predictive model due to the nonlinear nature of diffuse reflectance across different spatial frequencies and wavelengths. However, the use of more complex algorithms may obscure the model’s interpretability.

Applying this two-step framework to clinical studies necessitates access to biopsy samples, which are typically unavailable under standard protocols, as noted in the study limitations section above. Importantly, the framework is adaptable to any continuous metric of burn severity, such as burn depth measured via polarization-sensitive optical coherence tomography (PS-OCT).^93^^,^^94^ In this context, PS-OCT data are acquired from a small ROI, functioning as an optical biopsy, and used to inform the SFDI-based model in generating large-FOV maps for burn severity assessment. Moreover, the framework can be extended to clinician-performed burn depth grading. Although linear regression is traditionally suited for continuous variables, it can be repurposed for classification by mapping categorical grades to numerical values (e.g., 0 to 4 for unburned skin to FT burns). A key limitation, however, is the reliance on physician judgment, which may not always accurately reflect the true severity of the burn and, in fact, is one of our motivations for developing a more objective means for assessment of burn severity.

Finally, we demonstrate the use of MLR as a straightforward and interpretable analysis method that enables SFDI in burn severity assessment. The model’s interpretability facilitates the identification of key features (i.e., the optimal combinations of spatial frequencies and wavelengths). These interpretability advantages, combined with the clinical insights gained from our CDD threshold analysis, position this framework as a promising foundation for translational burn assessment technologies, including the development of a more compact and efficient system.

## 6 Conclusion

This study successfully established SFDI as a viable tool for quantitative burn severity assessment through our two-step regression framework. Building on the clinical insights discussed above, we developed a regression analysis approach utilizing SFDI calibrated reflectance ($R_{d}$) to assess histology-derived CDD and healing outcome as direct and indirect metrics of burn wound severity. Our analysis suggests that MLR can yield a predictive model of day-1 CDD from input same-day SFDI $R_{d}$ maps with a mean error of 105  μm and an adjusted $R^{2}$ of 0.71 through all folds of LOSO CV. Implementation of LogR yielded a probabilistic model of day-28 healing outcome using day-1 CDD. This probabilistic model was then used to classify individual pixels into “heal” or “no heal” using a Youden J statistic of 0.55, corresponding to a probability threshold of 0.31 and ∼181  μm in CDD. ROC curve analysis showed a mean AUC of 0.81 for the training data and 0.88 for the testing data across all folds of LOSO CV. We also demonstrated that histology-informed day-1 CDD can predict day-28 healing outcome with a true positive rate (i.e., sensitivity) of 0.79 and a true negative rate (i.e., specificity) of 0.76 at the maximum Youden J statistics value of 0.55. Overall, we demonstrated that our two-step regression framework offers a predictive model of burn severity measured by day-1 CDD and day-28 healing outcomes using day-1 SFDI $R_{d}$ maps. The interpretability of this streamlined regression pipeline facilitates the identification of critical SFDI features for burn severity assessment, ultimately guiding the design of more compact systems.

## 7 Appendix A—Mathematical Terminology, Notations, and Definitions

### 7.1 Multiple Linear Regression

MLR is a method for deriving a linear equation that describes the relationship between multiple predictor variables and a single response variable.

### 7.2 Ordinary Least Squares

OLS is a method of finding regression coefficients that achieve the best-fit MLR line by minimizing the squared error of the predicted response variables. The mathematical definition of the optimization problem for N observations with P features is as follows:

$${\arg\;\min}_{\beta }(\overset{N}{\underset{j=1}{\sum }}(y-\overset{P}{\underset{i=1}{\sum }}\beta_{i}x_{i}-\beta_{0}{)}_{j}^{2}),$$

is the regression coefficient, x is the predictor variable, Y is the response variable, $\beta_{0}$ is the y-intercept, i is the feature index, and j is the observation index. In this study, “feature” refers to the SFDI calibrated reflectance at a known spatial frequency and wavelength. We implemented OLS for MLR using a MATLAB function called “regress”.

### 7.3 Root Mean Square Error

We use the RMSE of the predicted response variable to evaluate the performance of the linear models. RMSE is calculated for N observation as follows:

$$\mathrm{RMSE}=\sqrt{\frac{1}{N}\sum (y_{p}-y_{t}{)}^{2}},$$

$y_{p}$ is the predicted response variable, $y_{t}$ is the observed true response variable. In this study, RMSE is calculated by assessing errors of the complete model and not just the fitted line. This means that the RMSE calculation will account for the nonnegative boundary condition as discussed below. It should be noted that RMSE has the same unit as the response variable.

### 7.4 Adjusted Coefficient of Determination (Adjusted $R^{2}$)

We assess the linear fit using the adjusted coefficient of determination (Adj. $R^{2}$). The Adj. $R^{2}$ is calculated for N observations and N features as follows:

$$\mathrm{Adj.}\;R^{2}=1-(\frac{N-1}{N-P})\frac{\sum (y_{p}-y_{t}{)}^{2}}{\sum (y_{t}-\overset{¯}{y_{t}}{)}^{2}},$$

$y_{p}$ is the predicted response variable, $y_{t}$ is the observed true response variable. Unlike the unadjusted $R^{2}$, which typically increases with an increasing number of predictor variables, the Adj. $R^{2}$ assesses the model’s fitting performance regardless of the number of features used. This allows us to directly compare models following the feature selection step.

### 7.5 Nonnegative Boundary Condition

In this study, we chose CDD as the response variable of our MLR models. Thus, the values of CDD should remain nonnegative. Nonnegative boundary conditions refer to the practice of limiting the lower bound of predicted CDD to zero. We employed this condition after achieving a fitted MLR model. Subsequently, RMSE is calculated after the application of the nonnegative boundary condition, whereas the Adj. $R^{2}$ is assessed before such application of the condition.

### 7.6 Logistic Regression

LogR is a simple classification algorithm that utilizes fitting a log-based link function to a binary class A to generate a probability curve. In this study, we used the logit link function (i.e., $\log(\frac{\mu }{1-\mu })$ where μ is the probability of being classified as A). From the generated probability curve, a threshold can be chosen to classify input data points as A or not A. We implemented LogR using a MATLAB function called “fitglm” with the logit link function for a binomial distribution.

### 7.7 Receiver Operating Characteristic Curve and Youden’s J Statistic

Receiver operating characteristic (ROC) curves are used to assess the accuracy of classification algorithms. In the ROC curve, the true positive rate (i.e., sensitivity) is plotted against the false positive rate (i.e., 1—specificity). A perfect binomial distribution or probabilistic model yields an area under the curve (AUC) of 1. Furthermore, to determine an optimal threshold for the binary classification, one can determine a threshold that maximizes the Youden’s J Statistic defined as follows:

J=True Positive Rate−False Positive Rate=Sensitivity+Specificity−1.

In essence, maximizing Youden’s J Statistic finds the threshold that allows for the best balance of sensitivity and specificity. A Youden J value of 1 indicates a perfect model for both sensitivity and specificity, whereas a value of 0 indicates random chance. We implemented the ROC curve analysis using a MATLAB function called “perfcurve”.

### 7.8 Leave-One-Subject-Out Cross-Validation

In statistics and ML model development, cross-validation (CV) refers to the process of assessing the model’s performance by dividing the available data into training and testing sets. Leave-one-subject-out (LOSO) is a rigorous cross-validation method that treats the data from one subject as the testing set while the rest of the data is used for training. For each fold of the process, LOSO CV uses independent data (i.e., different subjects) for testing and thus fully captures the model’s performance behavior. In this study, “subject” refers to one of the four animals. Following LOSO CV, we used the data from all subjects to generate the “general” model.

## 8 Appendix B—Supplementary Information

### 8.1 Histology and Biopsy Details

Biopsies were fixed in 10% neutral buffered formalin for 48 h, stored in 70% ethanol, embedded in paraffin, sectioned into 6-μm-thick slices, deparaffinized, rehydrated with Histo-clear, and stained with PSR for 1 h. Samples underwent an acetic acid wash and were mounted in Cytoseal for imaging.

Parallel (0 deg) and cross (90 deg) polarized images were captured using a Jigtech polarization package by Daitron (N11599-00R-002, Abcam, Cambridge, United Kingdom) and a Keyence BZX microscope (Keyence, Osaka, Osaka, Japan). We acquired histological images at 2x magnification with fixed exposure time.

### 8.2 Animal Experiment Details

All procedures were carried out under UCI IACUC protocol AUP‐24‐056. Animal anesthesia procedures: For each subject, sedation was initiated with an intramuscular cocktail of Telazol (10  mg/kg) and Xylazine (4  mg/kg). Enrofloxacin (7.5  mg/kg) was administered intramuscularly as an antibiotic following sedation. The animals were intubated and anesthetized with isoflurane (1% to 3%) to maintain a tidal volume of 10  mL/kg, respiratory rate of 12 breaths/min, and inverse ventilation ratio of 1:2. Ringer’s lactate (5  mL/kg/h) was delivered intravenously through the lateral ear vein, and vital signs (heart rate, respiratory rate, end-tidal CO2, tidal volume, and oxygen saturation) were monitored to ensure proper anesthetic depth and animal well-being.

Animal wound care procedures: Buprenorphine (0.005 to 0.01  mg/kg) was administered intramuscularly ∼20  min before the end of the procedure and every 12 h as needed afterward. Postoperative checks were performed twice daily. Wounds were dressed with saline-soaked Telfa pads, secured with Loban dressing and a netting tube. Dressings were changed daily, with Telazol/Xylazine used to induce sedation during redressing. Animals were fasted before any procedure requiring sedation. On day 28, animals were euthanized following final imaging and biopsy procedures. Table 4 below describes the weight and sex of the animals used in the experiment. Table 5 also provides the complete list of all SFDI features (i.e., spatial frequencies and wavelengths) used in the MLR model and their associated regression coefficients (i.e., MLR beta) and p-values.

**Table 4:** Weight and sex of animals used in the experiment.

Animal	Sex	Weight [kg]
P1	Female	64
P2	Male	48.5
P3	Female	54
P4	Male	45

**Table 5:** Complete list of all SFDI features and their associated regression coefficients and t-statistic p-values from the general MLR model.

Feature number	Center Wavelength [nm]	Spatial frequency $\left[{\mathrm{mm}}^{-1}\right]$	MLR Beta	P-value
1	471	0	456.0141	2.00E-10
2	471	0.05	−324.056	0.003769
3	471	0.1	864.5977	0
4	471	0.15	−578.95	2.00E-10
5	471	0.2	−831.977	0
6	526	0	2036.755	0
7	526	0.05	268.7599	0.016859
8	526	0.1	−569.187	3.73E-07
9	526	0.15	−2122.66	0
10	526	0.2	294.5245	0.000449
11	591	0	1500.212	0
12	591	0.05	−1232.84	0
13	591	0.1	667.0291	9.04E-08
14	591	0.15	−474.19	1.85E-05
15	591	0.2	1384.186	0
16	621	0	−3080.84	0
17	621	0.05	−275.035	0.028404
18	621	0.1	269.6431	0.026138
19	621	0.15	1575.633	0
20	621	0.2	−729.742	0
21	659	0	287.6229	0.001019
22	659	0.05	−435.279	3.82E-05
23	659	0.1	−923.003	0
24	659	0.15	−495.198	6.59E-05
25	659	0.2	−292.751	0.007475
26	691	0	1036.49	0
27	691	0.05	1224.802	0
28	691	0.1	1327.772	0
29	691	0.15	−64.2697	0.44199
30	691	0.2	437.8722	1.20E-09
31	731	0	284.5767	0.003917
32	731	0.05	−758.251	1.00E-09
33	731	0.1	−948.293	0
34	731	0.15	410.4658	0.000762
35	731	0.2	1171.562	0
36	851	0	−831.182	0
37	851	0.05	−1078.07	0
38	851	0.1	297.5352	0.004559
39	851	0.15	217.7298	0.031422
40	851	0.2	−129.607	0.087625
41 (constant)			982.1283	

### 8.3 All Outputs From MLR and LogR Models

**Fig. 9:**
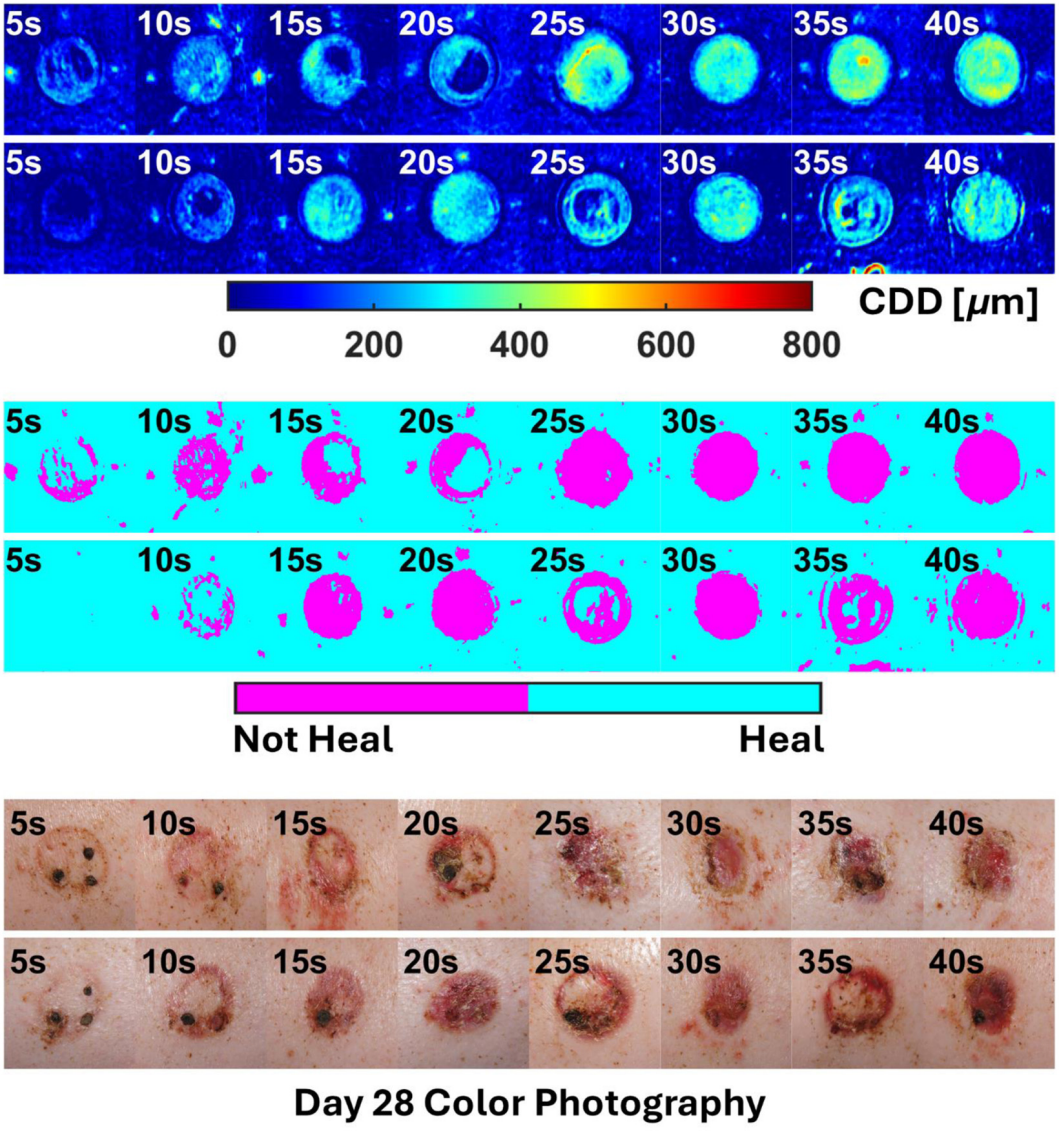
All outputs for subject P1.

**Fig. 10:**
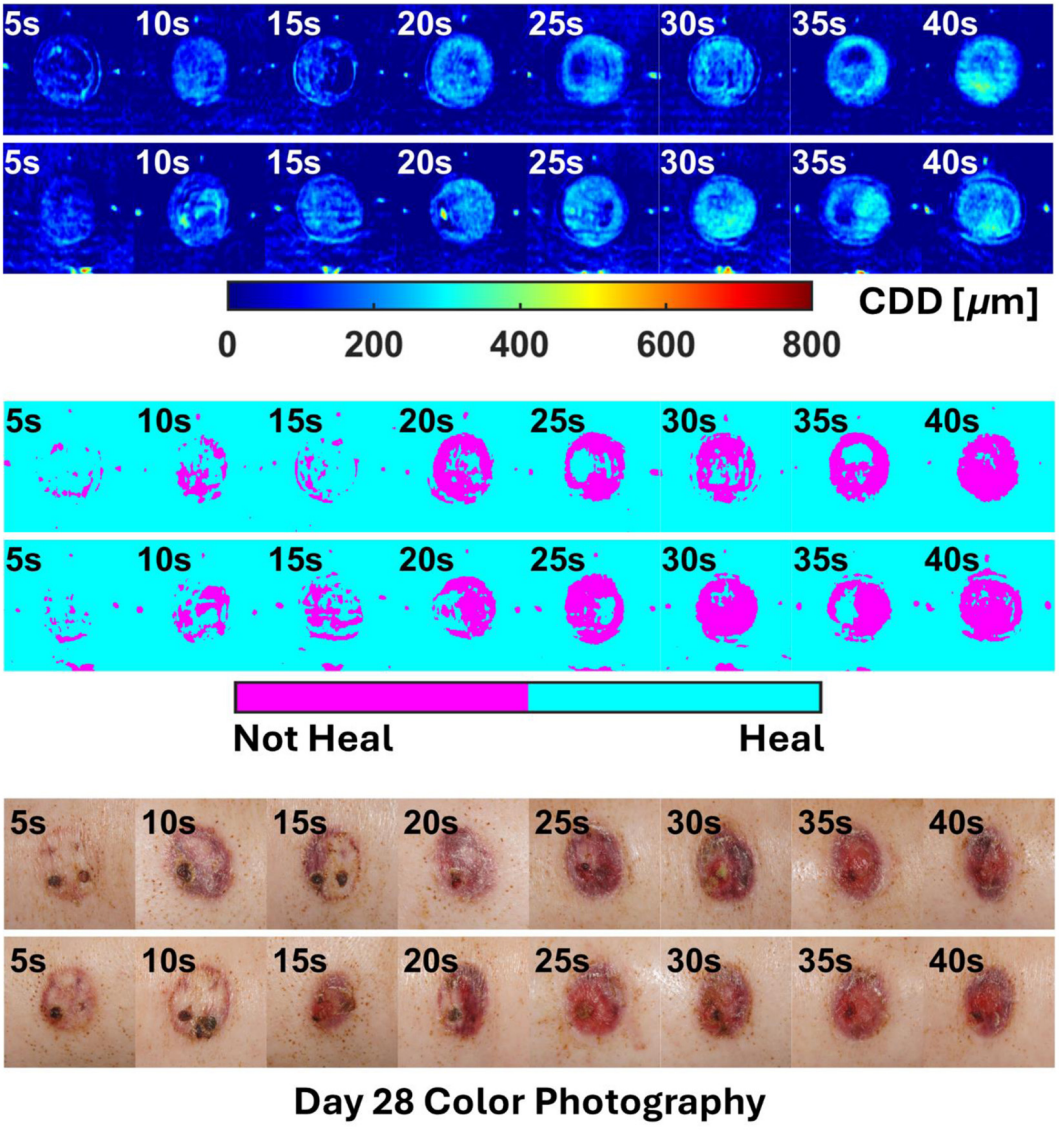
All outputs for subject P2.

**Fig. 11:**
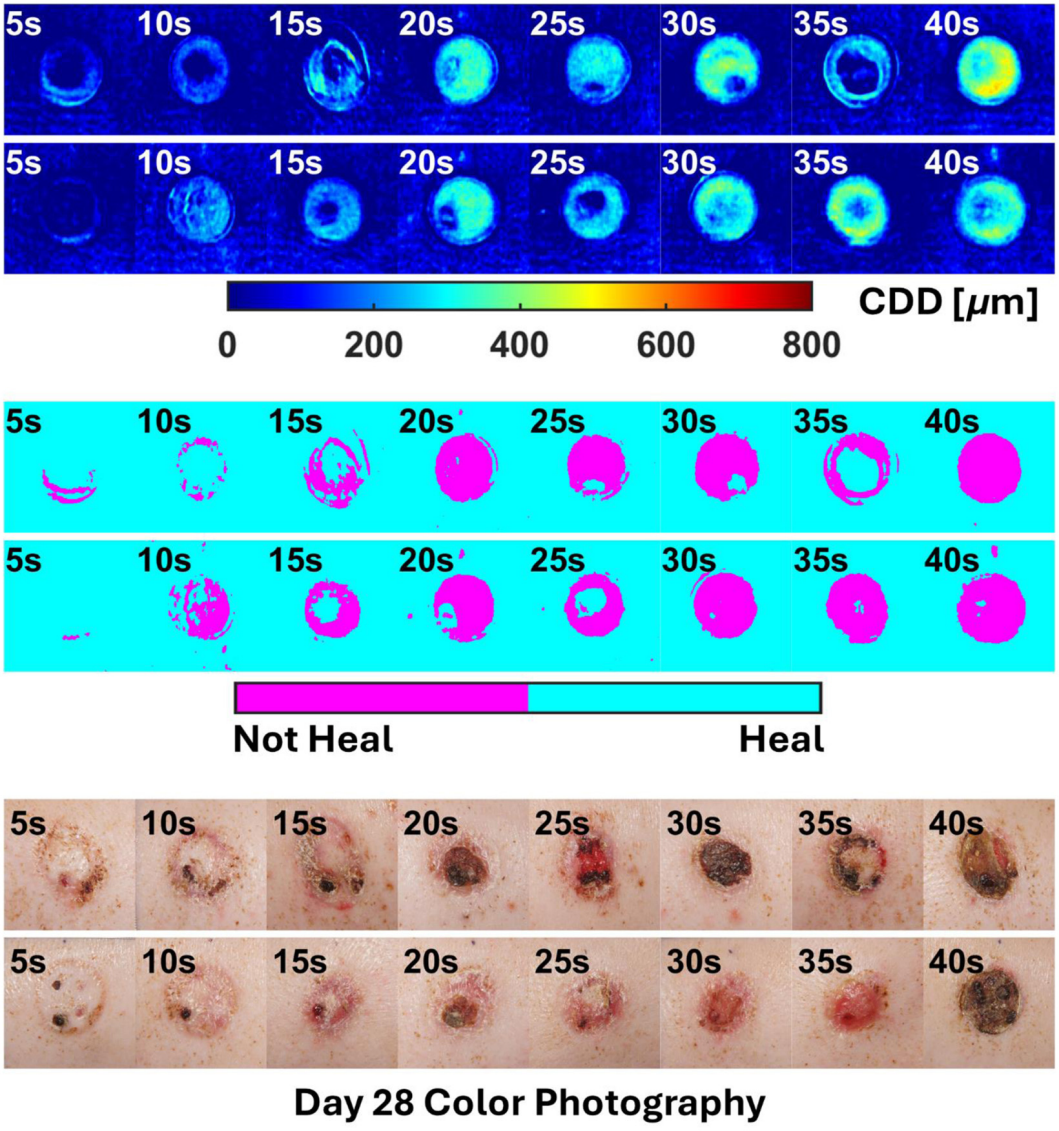
All outputs for subject P3.

**Fig. 12:**
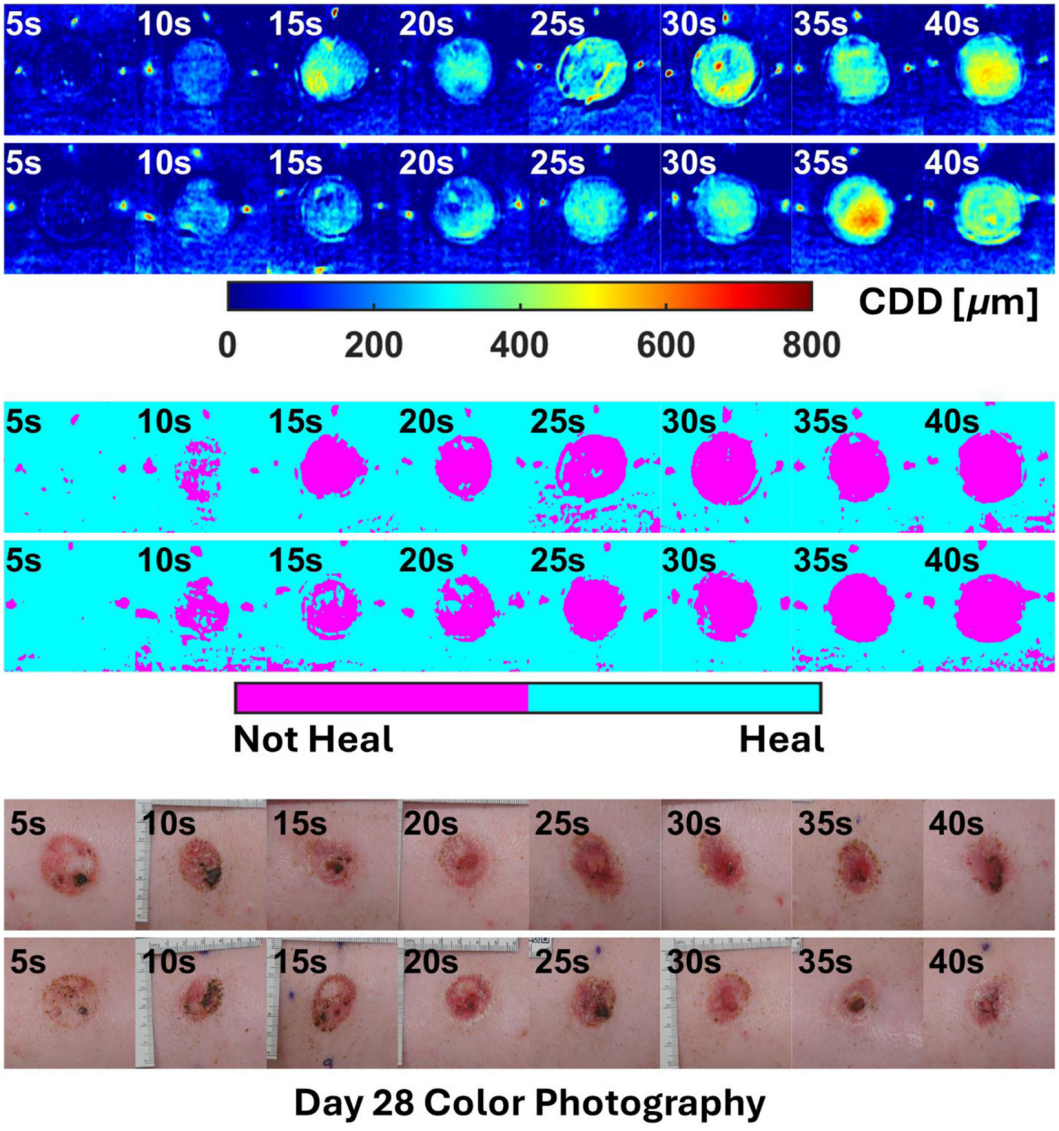
All outputs for subject P4.

The overall results of the two-step regression pipeline (MLR + LogR) for all animals are shown in Figures 9–12.

## Data Availability

The datasets generated and/or analyzed during the current study are available upon reasonable request.
